# Additive interaction of family medical history of diabetes with hypertension on the diagnosis of diabetes among older adults in India: longitudinal ageing study in India

**DOI:** 10.1186/s12889-024-18146-0

**Published:** 2024-04-10

**Authors:** Waquar Ahmed

**Affiliations:** School of Health Systems Studies, Tata Institute of Social Sciences, Mumbai, India

**Keywords:** Additive interaction, Synergistic effect, Family medical history, Hypertension, Diabetes, Older adults, India

## Abstract

**Background:**

The present study aimed to estimate the additive interaction of family history of diabetes and hypertension on the diagnosis of diabetes among individuals aged 45 years and above in India. The coexistence of these two exposures may act synergistically on the risk of diabetes, leading to adverse health outcomes.

**Methods:**

The study utilized the data from the Longitudinal Ageing Study in India (LASI) Wave 1 (2017–2018). The total sample size for the current study was 58,612 individuals aged 45 years and above. Multivariable logistic regression models were employed to determine the individual and joint effect of a family history of diabetes with hypertension on diabetes. An additive model was applied to assess the interaction effect of the family medical history of diabetes with hypertension on the diagnosis of diabetes by calculating three different measures of additive interaction such as the relative excess risk due to interaction (RERI), attribution proportion due to interaction (AP), and synergy index (S).

**Results:**

The prevalence of diabetes was three times higher among individuals with family history of diabetes (27.8% vs. 9.2%) than those without family history. Individuals with family history of diabetes (AOR: 2.47, CI: 2.11 2.89) had 2.47 times higher odds of having diabetes than those without family history. The prevalence of diabetes was significantly higher among individuals with hypertension and family history of diabetes (46.6%, 95% CI: 39.7–53.6) than those without the coexistence of family history of diabetes and hypertension (9.9%, 95% CI: 9.5–10.4), individuals with hypertension and without a family history of diabetes (22.7%, 95% CI: 21.2–24.2), and individuals with family history of diabetes and without hypertension (16.5%, 95% CI: 14.5–18.7). Moreover, the adjusted odds ratio (AOR) of the joint effect between family medical history of diabetes and hypertension on diabetes was 9.28 (95% CI: 7.51–11.46). In the adjusted model, the RERI, AP, and S for diabetes were 3.5 (95% CI: 1.52–5.47), 37% (0.37; 95% CI: 0.22–0.51), and 1.69 (95% CI: 1.31–2.18) respectively, which indicates that there is a significant positive interaction between family history of diabetes and hypertension on the diagnosis of diabetes. The study findings on interaction effects further demonstrate consistent results for two models of hypertension (self-reported hypertension and hypertensive individuals receiving medication) even after adjustment with potential confounding factors on diabetes (self-reported diabetes and individuals with diabetes receiving medication).

**Conclusions:**

The study findings strongly suggest that the interaction of family history of diabetes with hypertension has a positive and significant effect on the risk of diabetes even after adjustment with potential confounding factors. Furthermore, the findings indicate a synergistic effect, emphasizing the importance of considering both family medical history of diabetes and hypertension when assessing diabetes risk and designing preventive strategies or interventions.

## Background

Diabetes is a heritable disease. Evidences from twin or family based studies have demonstrated the estimated heritability ranging from 30 to 70% depending on the age at onset [1–3]. Additionally, the strongest heritability for type 2 diabetes was observed in patients with age at onset between 35 and 60 years [1]. Family history of diabetes is a well-established and independent risk factor for developing diabetes [4–6], In addition, family medical history represents valuable genomic information that can be used to understand the complex interplay of environmental, behavioral, and genetic factors that contribute to its development [4, 6–9]. Moreover, a previous study demonstrated that that individuals with diabetes had an adjusted prevalence ratio of 4.27 for family medical history of diabetes than those without diabetes or prediabetes [10].

Extensive genome-wide genetic research on prevalent diabetes in large cohorts of adult populations has shown the presence of more than 500 genetic variants that demonstrate associations with diabetes [11]. Additionally, numerous genetic variants have been identified that elevate the risk of coronary artery disease (CAD) [12] in individuals with diabetes and also exhibit the association with diabetic end-organ complications, such as retinopathy [13], nephropathy [14], and neuropathy [15]. Further, existing literature suggests that beyond its effect on the risk of developing diabetes, possessing a family medical history of diabetes independently heightens the likelihood of having vascular complications, notably coronary heart disease (CHD) and stroke [5].

Hypertension has long been known to be associated with increased risk of developing diabetes [16–19] and occurs in roughly 50–80% of individuals with type 2 diabetes [20]. One prior study demonstrated that among people without diabetes, hypertension at baseline was a strong predictor of developing diabetes over time. Moreover, incidence of hypertension was found to be significantly increased in people with diabetes [21]. Diabetes and hypertension are frequently co-occurring conditions, reflecting the significant overlap in their underlying causes and biological mechanisms [22]. In addition, a substantial portion of individuals diagnosed with diabetes demonstrate inadequately controlled hypertension [21]. In patients with diabetes, hypertension significantly increases the risk of cardiovascular disease [23].

Family history is a significant indicator of genetic factors, and it is frequently employed as an alternative measure to investigate the association between genetic factors and diseases [24–27]. Moreover, despite the recent identification of numerous genetic variants associated with type 2 diabetes, most of these variants have small effect sizes and cannot fully explain the effect of family history as an independent risk factor on the risk of type 2 diabetes [5, 10, 28]. Similarly, relying solely on hypertension as a predictor of individual risk of diabetes is insufficient. Hereditary factors may elucidate why some specific hypertensive individuals are more susceptible to diabetes. A prior study demonstrated that individuals with hypertension, exhibited a notably elevated prevalence (41.76%) of a familial history of diabetes in comparison to those without hypertension [10]. Given the higher prevalence, their coexistence may act synergistically on the risk of diabetes. There is a substantial gap in the literature concerning the interaction effect of family medical history of diabetes with hypertension on diabetes in low and middle-income countries (LMICs), especially in India. The current study aimed to assess the additive interaction of family medical history of diabetes with hypertension on diabetes. Understanding the synergistic effect is imperative for developing effective risk assessment, prevention, and management strategies and interventions.

## Methods

### Data

**Fig. 1:**
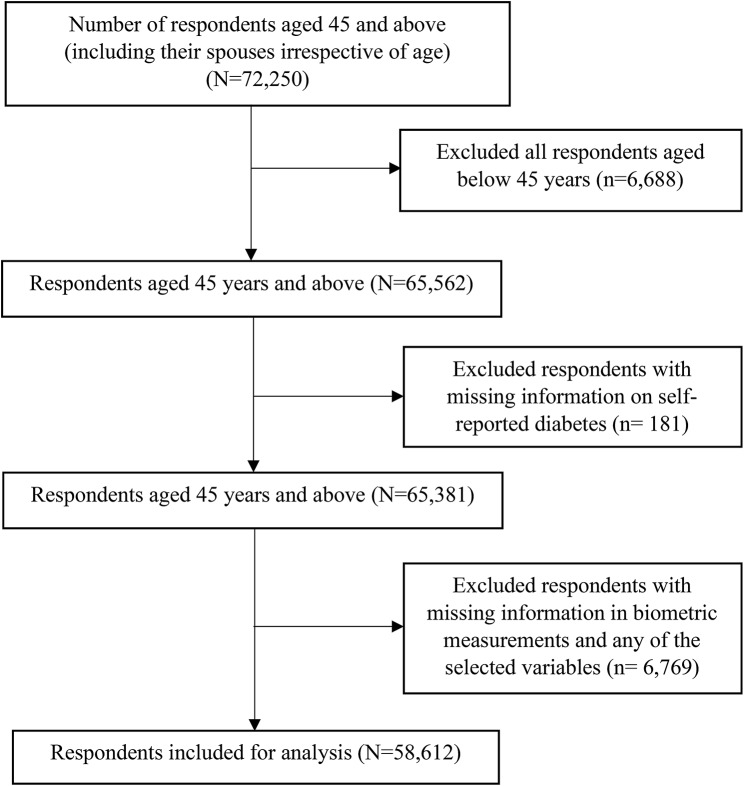
Study sample flowchart

The data from the Longitudinal Ageing Study in India (LASI) Wave 1 (2017–2018) were used in this study. The survey collected data on the health, economic, and social factors, and consequences of India’s population ageing. The LASI is a full-scale, nationally representative survey that included 72,250 individuals aged 45 years and older and their spouses (irrespective of age) across all states and union territories (UTs) of India except Sikkim. The LASI uses a multistage stratified area probability cluster sampling to select the eventual units of observation. This study presents scientific evidence on chronic health conditions, biomarkers, symptom-based health conditions, and functional and mental health. The LASI survey was conducted with a three-stage sampling design in rural areas and a four-stage sampling design in urban areas. In each state/UT, in the first stage, Primary Sampling Units (PSUs) were selected, and in the second stage, villages in rural areas and wards in urban areas were selected in the selected PSUs. In the third stage, households were selected from each selected village; however, sampling in urban areas involved an additional stage, i.e., the random selection of one Census Enumeration Block (CEB) in each urban area. In the fourth stage, households were selected from each CEB. The main goal was to select a representative sample at each stage of sample selection. The detailed methodology and extensive information on the survey’s design and data collection are available in the report [29]. The present study is based on 65,562 respondents aged 45 years and above excluding those less than 45 years (*n* = 6,688). Additionally, after removing respondents with missing information on self-reported diabetes (*n* = 181) and those with incomplete information in any of the selected variables and biometric measurements (*n* = 6,769) (including family history: 394; hypertension: 2; physical inactivity: 53; ADL: 121; IADL: 180; biometric measurements of body mass index: 6,328 and any selected variables), the total sample size for the analysis was 58,612 respondents. (Fig. 1 presents the inclusion and exclusion criteria for the study sample.

## Measures

### Outcome variables

The main outcome variable was self-reported diabetes. In the study, respondents were asked, “Has any health professional ever diagnosed you with diabetes?”. The responses were coded as no and yes. In LASI, respondents were asked additional questions to those who reported being diagnosed with a disease by a medical professional, including the diagnosing physician, the date of diagnosis, and whether they are currently receiving treatment. Participants were asked, “In order to treat or control your diabetes or high blood sugar, are you currently taking medications?”. Interaction effects were estimated in two different study sample, (1) self-reported diabetes and (2) Individuals with diabetes, who were taking treatment.

### Key explanatory variable

The main explanatory variable was self-reported hypertension, and family medical history of diabetes. In the study, respondents were asked, “Has any health professional ever diagnosed you with high blood pressure or hypertension?”. The responses were coded as no and yes. In the LASI, to understand the genetic risk factors for diabetes, information was collected about the respondent’s family medical history; the family medical history of the father, mother, brother, and sister were selected for the analysis.

In LASI, respondents were asked additional questions concerning medication to those who reported being diagnosed with a disease by a medical professional including whether they are currently receiving treatment. Participants were asked, “In order to control your blood pressure or hypertension, are you currently taking any medication?”.

### Other covariates

Age was coded as 45–54 years, 55–64 years, 65–74 years, and 75 + years. Gender was coded as male and female. Education was recoded as no education, primary, secondary, and higher. Marital status was coded as currently married, widowed, and others, not in a union. Working status was coded as never worked, currently working, and currently not working. Alcohol use was coded as ‘no’ and ‘yes’; smoking and chewing tobacco were coded as ‘never’, ‘former, and ‘current’ The BMI was computed by dividing the weight (in kilograms) by the square of the height (in meters). BMI was coded according to the criteria of the World Health Organisation’s classification; as underweight (< 18.5 kg/m2), normal weight (18.5–24.9 kg/m2), overweight (25.0–29.9kg/m2), and obesity (≥ 30.0 kg/m2), for the analysis, overweight and obesity were combined [30]. The monthly per capita expenditure quintile (MPCE) or consumption quintile was categorized into five quintiles, poorest, poor, middle, rich, and richest. Religion was categorized as Hindu, Muslim, Christian, and Others. The social group (caste) was categorized as Scheduled Castes (SC), Scheduled Tribes (ST), Other Backward Classes (OBC), and others. The ‘other’ category in caste is identified as non SC/ST and OBC. The place of residence was coded as urban and rural. The regions were categorized as North, Central, East, Northeast, West, and South. In the study, respondents were asked, “Has any health professional ever diagnosed you with high cholesterol”. The responses were coded as no and yes.

To assess difficulty in activities of daily living (ADL), respondents were asked questions on the following six activities: difficulty in dressing, walking across a room, bathing, eating, getting in or out of bed, and using the toilet. Individuals who reported difficulty with any activity for more than three months were coded as “yes” and otherwise “no”.

To assess difficulty in instrumental activities of daily living (IADL), respondents were asked if they had difficulty in performing in any of the following seven activities: preparing hot meals, shopping for groceries, making telephone calls, taking medication, doing household work, managing finances, and getting around or finding an address in an unfamiliar location (Alpha value: 0.88). Those who reported trouble with any of these activities for more than three months were labelled “having difficulty.” Otherwise, they were categorized as having “no difficulty.” ADL and IADL are considered as measures of functional health and a prolonged difficulty in any of the items refers to individuals’ dependence on others and/or instrumental devices.

To assess the level of physical activities, participants were asked about the type and amount of physical activity integrated into daily life. For vigorous activities, participants were asked “How do you often take part in sports or vigorous activities such as running or jogging, swimming, going to the health centre/gym, cycling, digging with a spade or shovel, heavy lifting, chopping, farm work, fast bicycling, and cycling with loads?”. For moderate activities, participants were asked, “How do you often take part in sports or activities that are moderately energetic such as cleaning house, washing clothes by hand, fetching water, or wood, drawing water from a well, gardening, bicycling at a regular pace, walking at a moderate pace, dancing, floor or stretching exercises?”. The available responses for evaluating moderate and vigorous activities were as follows: every day, more than once a week, once a week, one to three times per month, and hardly ever or never. For both moderate and vigorous activities, participants were also asked “On the days you did the activity, how much time did you usually spend doing any activity?”.

Weekly durations of both moderate and vigorous physical activities were computed: Moderate physical activity was defined as those who engaged in a minimum of 150 min of moderate-intensity physical activity in a week, while vigorous physical activity encompassed those who engaged in a minimum of 75 min of vigorous-intensity physical activities in a week. Respondents were subsequently categorized into two groups based on their engagement in moderate and vigorous activities: “Physically active,” denoting those who engaged more than once a week, and “Physically inactive,” characterizing individuals who engaged once a week or less often. Subsequently, a binary variable of physical activity variable was created as “Physically active,” comprising those engaged in either moderate or vigorous physical activities, and otherwise “Physically inactive,” [31, 32].

### Statistical analysis

Multivariable logistic regression was used to calculate the unadjusted and adjusted estimates, aiming to evaluate the joint effect of family medical history of diabetes with hypertension on diabetes. Further, an additive model was employed to assess the additive interaction effect of family medical history of diabetes with hypertension on diabetes by estimating three distinct measures of additive interaction: the relative excess risk due to interaction (RERI), attribution proportion due to interaction (AP), and synergy index (S). Interaction on an additive scale means that the combined effect of two exposures is not simply the sum of their individual effects. Instead, the two exposures interact with each other to produce a combined effect that is greater (or smaller) than the sum of their individual effects [33, 34].

The interaction measures on the additive scale are defined as RERI (excess risk of the outcome due to the interaction between two factors) = OR11 - OR10 - OR01 + 1; AP (proportion of the combined effect that is attributable to the interaction between two factors) = RERI / OR11; S (ratio between combined effect and individual effects) = (OR11–1) / (OR10–1) + (OR01–1). RERI = 0 indicates no interaction, RERI > 0 suggests positive interaction, RERI < 0 suggests negative interaction, AP = 0 suggests no interaction, AP > 0 suggests positive interaction, AP < 0 suggests negative interaction, S = 1 suggests no interaction, S > 1 suggests positive interaction, S < 1 indicates negative interaction [33–35].

Bivariate analysis was performed to investigate the prevalence of diabetes and proportion of individuals who were using medication in relation to selected variables. A chi-square test and bivariate analysis were also employed to investigate the prevalence of diabetes concerning the combined effect of a family medical history of diabetes with hypertension. Moreover, multivariable binary logistic regression analysis [36] was employed to establish the association between diabetes, and main explanatory variables including hypertension and family history of diabetes (comprising overall family history, parental, sibling, and specific histories related to the father, mother brother and sister).

In the current study, the multivariable logistic regression and additive interaction models were adjusted for potential confounding factors, including age, sex, education, working status, marital status, residence, MPCE, religion, caste, region, physical inactivity, smoking, chewing tobacco, alcohol consumption, body mass index (BMI), ADL, IADL, and high cholesterol. The survey weights were applied during the analysis to account for sample clustering and present population estimates. All the analyses were conducted using Stata version 14.1 [37].

## Results

Table 1 presents the sample characteristics and percentage distribution of diabetes and its treatment among individuals aged 45 and above. A proportion of 34.43% of the participants were 65 years and above. Approximately 54% of the sample population was female. About 50.45% of the sample had no education during the survey. A large proportion of the sample (73.93%) were in marital union during the survey. Further nearly 70% of the participants were living in rural areas.

**Table 1:** Sample characteristics and percentage distribution of diabetes and its treatment by background characteristics among individuals aged 45 years and above, Longitudinal Ageing Study in India (LASI, 2017-18)

Background Characteristics	Sample		Diabetes					Taking treatment for diabetes			
	N	w col %	N	w % (95% CI)				N			w row %
Sociodemographic variables											
**Age groups**											
45–54	21,774	35.10	1,994	8.1 (7.4,8.8)			1,607		80.47		
55–64	18,214	30.46	2,596	13.1 (12.2,14.2)			2,136		82.39		
65–74	13,002	24.01	2,126	16.0 (13.7,18.6)			1,800		85.15		
75+	5,622	10.42	759	11.8 (10.2,13.5)			626		79.42		
**Gender**											
Male	27,143	45.89	3,554	12.0 (11.2,12.7)		2,887			80.72		
Female	31,469	54.11	3,921	11.9 (10.7,13.1)		3,282			84.06		
**Education level**											
No education	27,496	50.45	2,284	7.8 (7.2,8.4)		1,797			76.89		
Primary	14,648	23.43	2,100	13.5 (12.5,14.7)		1,714			82.66		
Secondary	10,848	16.28	1,894	17.8 (14.7,21.5)		1,618			88.21		
Higher	5,620	9.84	1,197	19.3 (16.6,22.3)		1,040			85.23		
**Working Status**											
Never Worked	16,017	25.98	2,519	15.4 (13.2,17.9)		2,184			86.48		
Currently working	27,333	47.28	2,370	8.2 (7.5,8.9)		1,856			79.90		
Currently Not working	15,262	26.74	2,586	15.1 (14.2,16.0)		2,129			81.10		
**Marital Status**											
Currently married	43,934	73.93	5,640	11.7 (11.1,12.3)		4,660			82.35		
Widowed	12,805	23.24	1,615	12.8 (10.5,15.6)		1,328				83.00	
D/S/D/Others	1,873	2.82	220	9.6 (7.5,12.3)		181			82.58		
**Place of Residence**											
Rural	38,361	70.12	3,295		8.2 (7.8,8.6)	2,524				76.60	
Urban	20,251	29.88	4,180		20.7 (18.5,22.9)	3,645				88.02	
**Caste**											
Scheduled caste	9,872	19.42	982	8.5 (7.6,9.5)		794			78.71		
Scheduled tribe	10,255	8.63	745	4.7 (4.0,5.5)		554			72.82		
OBC	22,143	45.49	3,052		13.4 (12.0,15.0)	2,563				85.26	
Others	16,342	26.45	2,696		14.2 (13.4,15.0)	2,258				80.78	
**MPCE quintile**											
Poorest	11,537	20.98	1,018		8.6 (7.6,9.6)	804				78.84	
Poorer	11,851	21.28	1,233		9.2 (8.5,10.0)	979				77.56	
Middle	11,855	20.48	1,469		10.6 (9.6,11.6)	1,207				81.70	
Richer	11,826	19.68	1,721		14.5 (12.4,17.0)	1,439				84.00	
Richest	11,543	17.58	2,034		17.7 (15.2,20.5)	1,740				86.98	
**Region**											
North	10,799	12.71	1,312		10.6 (9.8,11.4)	1,095				83.18	
Central	8,059	21.09	561		7.1 (6.3,7.8)	418				72.59	
East	10,606	23.90	1,035		8.9 (8.2,9.7)	787				75.40	
Northeast	7,577	3.38	517		7.3 (6.4,8.3)	347				64.58	
West	7,701	15.94	1,171		13.6 (12.4,14.8)	986				80.67	
South	13,870	22.98	2,879		19.7 (17.1,22.7)	2,536				90.78	
**Religion**											
Hindu	43,025	82.50	5,269		11.6 (10.7,12.5)	4,338				82.42	
Muslim	6,932	11.04	1,125		13.3 (11.8,15.0)	939				81.14	
Christian	5,874	2.95	695		14.8 (11.9,18.3)	561				86.60	
Others	2,781	3.51	386		12.9 (11.2,14.8)	331				85.08	
**Lifestyle variables**											
**Smoking tobacco**											
Never	47,832	82.74	6,504		12.6 (11.7,13.5)	5,418				83.48	
Former	2,667	3.78	385		12.7 (10.9,14.8)	325				84.32	
Current	8,113	13.48	586		7.4 (6.6,8.4)	426				71.63	
**Chewing tobacco**											
Never	45,753	76.42	6,288		13.0 (12.1,14.0)	5,269				83.62	
Former	1,433	2.38	188		11.2 (9.0,13.7)	147				81.47	
Current	11,426	21.20	999		8.1 (7.4,8.9)	753				76.32	
**Alcohol Use**											
No	48,053	84.84	6,382		12.4 (11.5,13.3)	5,332				83.27	
Yes	10,559	15.16	1,093		9.3 (8.5,10.2)	837				76.91	
**Physical inactivity**											
Active	37,868	65.78	4,402		11.1 (10.1,12.2)	3,600				82.68	
Inactive	20,744	34.22	3,073		13.5 (12.7,14.2)	2,569				82.27	
**BMI categories**											
Normal	30,635	51.57	3,356		10.3 (9.7,10.9)	2,733				80.61	
Underweight	10,847	21.34	423	3.7 (3.2,4.2)		282			61.45		
Overweight/obese	17,130	27.09	3,696	21.5 (19.3,23.9)		3,154			87.09		
**Morbidities**											
**Difficulty in ADL**											
No	50,686	84.49	6,126		11.7 (10.8,12.6)	5,037				83.07	
Yes	7,926	15.51	1,349		13.2 (12.2,14.4)	1,132				79.88	
**Difficulty in IADL**											
No	39,612	63.49	4,830		11.1 (10.5,11.7)	3,954				81.80	
Yes	19,000	36.51	2,645		13.3 (11.7,15.1)	2,215				83.56	
**High Cholesterol**											
No	56,542	97.75	6,610		11.4 (10.6,12.2)	5,400				82.16	
Yes	2,070	2.25	865		34.6 (30.8,38.6)	769				87.68	
**Hypertension**											
No	41,881	73.02	2,918		6.2 (5.8,6.6)	2,393				81.36	
Yes	16,731	26.98	4,557		27.5 (25.4,29.7)	3,776				83.22	
Treatment for HT*											
No	4,659	28.17	679		14.2 (12.7,15.8)	312				39.78	
Yes	12,072	71.83	3,878		32.7 (30.0,35.5)	3,464				90.63	
**Treatment for HT**											
No	46,540	80.62	3,597		6.9 (6.5,7.3)	2,705				73.31	
Yes	12,072	19.38	3,878		32.7 (30.0,35.5)	3,464				90.63	
**Family Medical History**											
**Family History of Diabetes**											
No	49,790	85.67	4,857		9.2 (8.8,9.8)	3,893				80.33	
Yes	8,822	14.33	2,618		27.8 (24.1,31.8)	2,276				86.87	
**Parental FH**											
No	53,052	90.72	5,703		9.9 (9.5,10.5)	4,625				81.15	
Yes	5,560	9.28	1,772		31.1 (25.6,37.0)	1,544				86.80	
**FH of Father**											
No	56,011	95.71	6,561		10.8 (10.2,11.5)	5,376				82.05	
Yes	2,601	4.29	914		36.5 (28.7,45.2)	793				85.63	
**FH of Mother**											
No	55,037	94.24	6,331		10.9 (10.2,11.5)	5,162				81.60	
Yes	3,575	5.76	1,144		29 (22.8,36.1)	1,007				88.14	
**Sibling FH**											
No	54,378	93.19	6,172		10.4 (10.0,11.0)	5,018				80.75	
Yes	4,234	6.81	1,303		31.8 (25.5,39.0)	1,151				90.47	
**FH of Brother**											
No	55,572	94.92	6,503		10.7 (10.2,11.2)	5,303				80.84	
Yes	3,040	5.08	972		34.6 (26.5,43.7)	866				92.23	
**FH of Sister**											
No	56,840	97.24	6,857		11.2 (10.6,11.9)	5,615				81.69	
Yes	1,772	2.76	618		36.4 (26.3,47.7)	554				91.50	
**Total**	**58,612**	**100**	**7,475**		**11.9 (11.2,12.7)**	**6,169**				**82.52**	

*Notes*: w %: weighted percentages to account for survey design and to provide national population estimates; ADL: Activities of daily living; IADL: Instrumental activities of daily living; MPCE: Monthly per capita consumption expenditure; FH, family medical history of diabetes; HT, hypertension; *, treatment for hypertension among individuals with self-reported hypertension

Table 1 also depicts the prevalence of diabetes among adults aged 45 years and above. The overall prevalence of diabetes was 11.9% (95% CI: 11.2, 12.7) and 82.5% of the individuals with diabetes were taking treatment. The prevalence of diabetes (16.0%) was higher among individuals in 65–74 years of age group. The prevalence of diabetes was higher among individuals with higher education (19.3%) than no education (7.8%). Additionally, diabetes was more prevalent among individuals living in urban areas (20.7%) than those in rural areas (8.2%). The results show that the prevalence of diabetes was higher among physically inactive (13.5% vs. 11.1%), overweight/obese participants (21.5% vs. 10.3%) and individuals with high cholesterol (34.6% vs. 11.4%) than their counterparts.

Moreover, diabetes was more prevalent among individuals with self-reported hypertension (27.5% vs. 6.2%) than their counterparts. The prevalence of hypertension was higher among hypertensive individuals who were taking treatment (32.7% vs. 14.2%) compared with individuals with self-reported hypertension who were not taking treatment.

Furthermore, we found that the prevalence of diabetes was 3 times higher among individuals with family history of diabetes (27.8% vs. 9.2%) than those without family history. Similarly, parental and sibling history of diabetes had 3 times higher prevalence of diabetes (31.1% and 31.8%) compared with those without parental and sibling history of diabetes (9.9% and 10.4%). We observed that the prevalence of diabetes was higher among individuals with father (36.5% vs. 10.8%), mother (29.0% vs. 10.9%), brother (34.6% vs. 10.7%) and sister (36.4% vs. 11.2%) medical history of diabetes compared with those without medical history of diabetes.

**Fig. 2:**
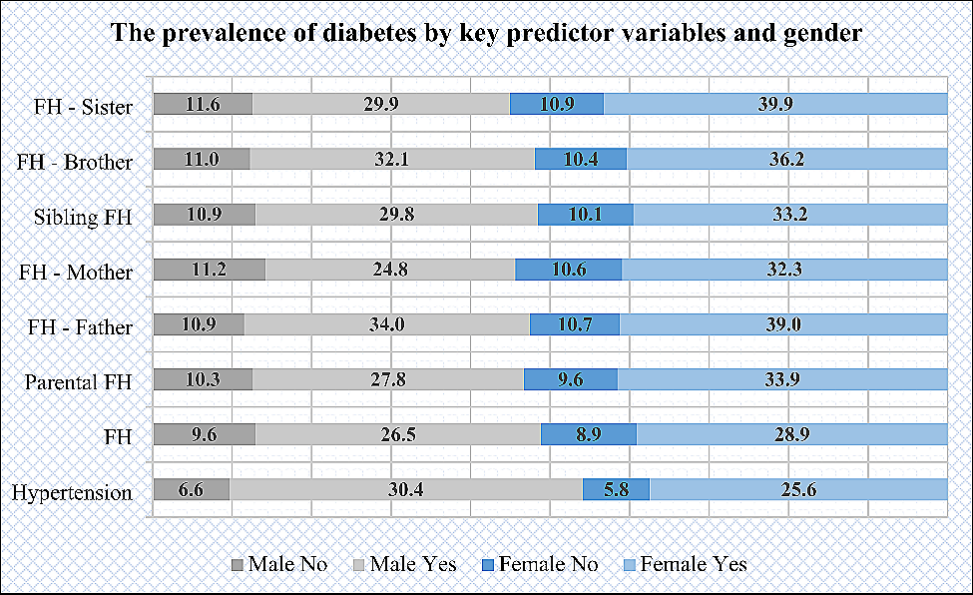
The prevalence of diabetes by key predictor variables and gender

**Fig. 3:**
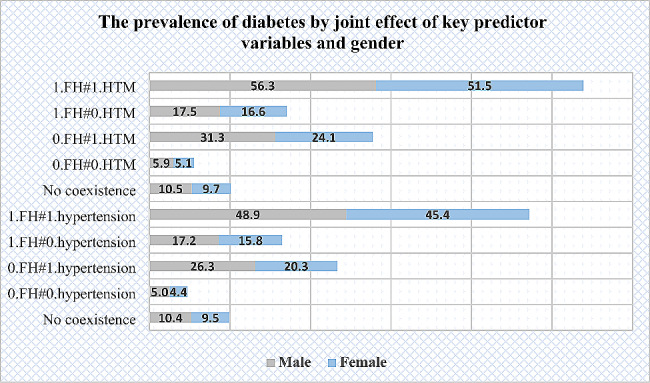
The prevalence of diabetes by joint effect of key predictor variables and gender HTM, hypertensive individuals who were taking medication; No-coexistence, either hypertension or family history of diabetes; FH, family history

Figure 2 illustrate that the prevalence of diabetes was higher among female participants with family history of diabetes, including parental, sibling, father, mother, brother, sister medical history of diabetes than males. Hypertension was more prevalent among male participants than females. On the other hand, Fig. 3 shows that the coexistence of family medical history and hypertension was higher among male participants than females.

Table 2 represents the prevalence of diabetes by key predictor variables among individuals aged 45 and above.

**Table 2:** The prevalence of diabetes by key predictor variables among individuals aged 45 and above

Diabetes						Taking Medication for diabetes					
Variables	N		Percentage (95% CI)		P-value	N		Percentage (95% CI)		P-value	
FH and Hypertension*					0.000					0.000	
No	5,922		9.9 (9.5,10.4)			4,806		8.0 (7.6,8.5)			
Yes	1,553		46.6 (39.7,53.6)			1,363		41.9 (34.6,49.5)			
FH and HT Medication*					0.000					0.000	
No	6,110		10.1 (9.6,10.6)			4,904		8.0 (7.6,8.5)			
Yes	1,365		53.1 (45.2,60.9)			1,265		50.4 (42.2,58.5)			
FH#Hypertension*					0.000					0.000	
0.FH#0.hypertension	1,853		4.7 (4.3,5.1)			1,480		3.8 (3.5,4.2)			
0.FH#1.hypertension	3,004		22.7 (21.2,24.2)			2,413		18.1 (16.7,19.6)			
1.FH#0.hypertension	1,065		16.5 (14.5,18.7)			913		13.5 (11.8,15.4)			
1.FH#1.hypertension	1,553		46.6 (39.7,53.6)			1,363		41.9 (34.6,49.5)			
FH#HT Medication*					0.000					0.000	
0.FH#0.HTM	2,344		5.5 (5.1,5.9)			1,694		3.9 (3.6,4.3)			
0.FH#1.HTM	2,513		26.9 (25.0,28.8)			2,199		23.7 (21.8,25.7)			
1.FH#0.HTM	1,253		17.0 (15.2,19.1)			1,011		13.0 (11.5,14.7)			
1.FH#1.HTM	1,365		53.1 (45.2,60.9)			1,265		50.4 (42.2,58.5)			
**Total**	**7,475**		**11.9 (11.2,12.7)**			**6,169**		**9.8 (9.1,10.6)**			

FH, family medical history of diabetes; HT, hypertension; HTM, hypertensive individuals who were taking medication

### The prevalence of diabetes based on the presence or absence of family medical history of diabetes and hypertension

#### Self-reported hypertension and family medical history of diabetes

The result presents that the prevalence of diabetes was significantly higher among individuals with hypertension and family history of diabetes (46.6%, 95% CI: 39.7–53.6) than those without the coexistence of family history of diabetes and hypertension (9.9%, 95% CI: 9.5–10.4), individuals with hypertension and without a family history of diabetes (22.7%, 95% CI: 21.2–24.2), individuals with family history of diabetes and without hypertension (16.5%, 95% CI: 14.5–18.7), and individuals without the presence of both family history of diabetes and hypertension (4.7%, 95% CI: 4.3–5.1).

#### Hypertensive individuals (taking antihypertensive medication) and family medical history of diabetes

The result presents that the prevalence of diabetes was significantly higher among hypertensive individuals (those who were taking medication for hypertension) with family history of diabetes (53.1%, 95% CI: 45.2–60.9) than those without the coexistence of family history of diabetes and hypertension (10.1%, 95% CI: 9.6–10.6), hypertensive individuals and without a family history of diabetes (26.9%, 95% CI: 25.0–28.8), individuals with family history of diabetes and without hypertension (17.0%, 95% CI: 15.2–19.1), and individuals without the presence of both family history of diabetes and hypertension (5.5%, 95% CI: 5.1–5.9).

### The prevalence of diabetes (who were taking medication) based on the presence or absence of family medical history and hypertension

#### Self-reported hypertension and family medical history of diabetes

The result presents that the prevalence of diabetes (those who were also taking medication) was significantly higher among individuals with hypertension with family history of diabetes (41.9%, 95% CI: 34.6–49.5) than those without the coexistence of family history of diabetes and hypertension (8.0%, 95% CI: 7.6–8.5), individuals with hypertension and without a family history of diabetes (18.1%, 95% CI: 16.7–19.6), individuals with family history of diabetes and without hypertension (13.5%, 95% CI: 11.8–15.4), and individuals without the presence of both family history of diabetes and hypertension (3.8%, 95% CI: 3.5–4.2).

#### Hypertensive individuals (taking antihypertensive medication) and family medical history of diabetes

The result presents that the prevalence of diabetes (those who were also taking medication) was significantly higher among hypertensive individuals (those who were taking medication for hypertension) with family history of diabetes (50.4%, 95% CI: 42.2–58.5) than those without the coexistence of family history of diabetes and hypertension (8.0%, 95% CI: 7.6–8.5), hypertensive individuals and without a family history of diabetes (23.7%, 95% CI: 21.8–25.7), individuals with family history of diabetes and without hypertension (13.0%, 95% CI: 11.5–14.7), and individuals without the presence of both family history of diabetes and hypertension (3.9%, 95% CI: 3.6–4.3).

Table 3 presents the unadjusted and adjusted logistic regression estimates for diabetes by family medical history of diabetes (overall, parental history, father, mother, sibling history, brother and sister) and hypertension among individuals aged 45 years and above. In the adjusted model, the results indicate that individuals with hypertension (AOR: 3.95, CI: 3.52–4.44) had 3.95 times higher odds of having diabetes than those without hypertension.

**Table 3:** Logistic regression estimates for self-reported diabetes and individuals with medication by family history and hypertension

	Diabetes		Taking Treatment for diabetes	
Background Characteristics	UOR 95%CI	AOR 95%CI	UOR 95%CI	AOR 95%CI
Hypertension				
No	Ref.	Ref.	Ref.	Ref.
Yes	5.77*** (5.07 6.56)	3.95*** (3.52 4.44)	5.61*** (4.84 6.51)	3.62*** (3.18 4.13)
**Family history of diabetes**				
No	Ref.	Ref.	Ref.	Ref.
Yes	3.78*** (3.09 4.62)	2.47*** (2.11 2.89)	3.97*** (3.18 4.96)	2.39*** (2.03 2.82)
**Parental history**				
No	Ref.	Ref.	Ref.	Ref.
Yes	4.08*** (3.11 5.36)	2.79*** (2.31 3.39)	4.20*** (3.11 5.68)	2.61*** (2.13 3.20)
**FH - Father**				
No	Ref.	Ref.	Ref.	Ref.
Yes	4.75*** (3.30 6.84)	3.29*** (2.46 4.41)	4.68*** (3.12 7.01)	2.95*** (2.18 3.99)
**FH - Mother**				
No	Ref.	Ref.	Ref.	Ref.
Yes	3.35*** (2.40 4.67)	2.16*** (1.71 2.73)	3.53*** (2.46 5.07)	2.10*** (1.64 2.69)
**Sibling history**				
No	Ref.	Ref.	Ref.	Ref.
Yes	4.00*** (2.92 5.50)	2.22*** (1.82 2.71)	4.39*** (3.10 6.22)	2.28*** (1.86 2.81)
**FH - Brother**				
No	Ref.	Ref.	Ref.	Ref.
Yes	4.41*** (3.00 6.49)	2.37*** (1.88 3.00)	4.95*** (3.26 7.51)	2.50*** (1.97 3.18)
**FH - Sister**				
No	Ref.	Ref.	Ref.	Ref.
Yes	4.53*** (2.82 7.27)	2.40*** (1.77 3.26)	4.95*** (2.96 8.27)	2.44*** (1.79 3.33)

*p < 0.05, **p < 0.01; ***p < 0.001; Ref, reference category; FH, Family history; UOR, Unadjusted odds ratio; AOR, Adjusted odds ratio, adjusted for age, sex, education, marital status, working status, residence, caste, MPCE, region, religion, smoking, chewing tobacco, alcohol, use, physical activity, body mass index, ADL, IADL, and high cholesterol

Our findings show that individuals with family history of diabetes (AOR: 2.47, CI: 2.11 2.89) had significantly 2.47 times higher odds of having diabetes than those without family history. Similarly, individuals with parental and sibling medical history had 2.79 and 2.22 times higher odds of having diabetes, respectively, than those without parental and sibling history. Moreover, our findings further demonstrate that individuals with father, mother, brother, sister medical history of diabetes had significantly 3.29, 2.16, 2.37, and 2.40 times higher odds of having diabetes, respectively compared with those without family history.

Table 4 provides the multivariable logistic regression estimates for diabetes by the joint effect of family history of diabetes and hypertension. The table also provides unadjusted and adjusted models of additive interaction of family history of diabetes with hypertension on diabetes. The current study provides estimates of the interaction effect on the additive scale for all models of hypertension (self-reported hypertension, and hypertensive individuals receiving treatments) in two different samples (self-reported diabetes and individuals with diabetes receiving medication).

**Table 4:** Additive interaction between family history of diabetes and hypertension on the diagnosis of diabetes among individuals aged 45 and above

	Model 1 (Unadjusted)	Model 2 (Adjusted)
**(1) Self-reported Diabetes**		
**(A) Self-reported HT**		
**Additive Interaction between family history and hypertension**		
0.FH#0.hypertension	Ref.	Ref.
0.FH#1.hypertension	5.93*** (5.25 6.70)	4.21*** (3.71 4.79)
1.FH#0.hypertension	4.00*** (3.37 4.78)	2.82*** (2.25 3.50)
1.FH#1.hypertension	17.63*** (13.13 23.68)	9.28*** (7.51 11.46)
**Measures of interaction on the additive scale (P-value, 95% CI)**		
**RERI**	**8.69**; 0.001 (3.61 13.77)	**3.5**; 0.001 (1.52 5.47)
**AP** ^$^	**0.49**; 0.000 (0.34 0.64)	**0.37**; 0.000 (0.22 0.51)
**S**	**2.09**; 0.000 (1.53 2.87)	**1.69**; 0.000 (1.31 2.18)
**(B)** ***Self-reported HT - taking medication***		
**Additive Interaction between family history and hypertension (taking medication)**		
0.FH#0.HTM	Ref.	Ref.
0.FH#1.HTM	6.34*** (5.60 7.18)	4.07*** (3.57 4.64)
1.FH#0.HTM	3.55*** (3.03 4.15)	2.60*** (2.14 3.16)
1.FH#1.HTM	19.57*** (14.12 27.12)	9.46*** (7.46 12.00)
**Measures of interaction on the additive scale (P-value, 95% CI)**		
**RERI**	**10.68**; 0.001 (4.37 17.01)	**3.79**; 0.000 (1.58 6.01)
**AP** ^$^	**0.55**; 0.000 (0.40 0.70)	**0.40**; 0.000 (0.25 0.55)
**S**	**2.35**; 0.000 (1.66 3.34)	**1.81**; 0.000 (1.37 2.40)
	**Model 1 (Unadjusted)**	**Model 2 (Adjusted)**
**(2) Self-reported diabetes, currently on medication**		
**(A)** ***Self-reported HT***		
**Additive Interaction between family history and hypertension**		
0.FH#0.hypertension	Ref.	Ref.
0.FH#1.hypertension	5.56*** (4.83 6.40)	3.73*** (3.23 4.31)
1.FH#0.hypertension	3.92*** (3.26 4.72)	2.53*** (1.98 3.23)
1.FH#1.hypertension	18.11*** (13.10 25.04)	8.50*** (6.82 10.61)
**Measures of interaction on the additive scale (P-value, 95% CI)**		
**RERI**	**8.68**; 0.001 (3.61 13.75)	**3.24**; 0.000 (1.46 5.03)
**AP** ^$^	**0.49**; 0.000 (0.34 0.64)	**0.38**; 0.000 (0.24 0.52)
**S**	**2.09**; 0.000 (1.53 2.87)	**1.76**; 0.000 (1.34 2.30)
**(B)** ***Self-reported HT - taking medication***		
**Additive Interaction between family history and hypertension (taking medication)**		
0.FH#0.HTM	Ref.	Ref.
0.FH#1.HTM	7.58*** (6.58 8.73)	4.74*** (4.08 5.49)
1.FH#0.HTM	3.65*** (3.07 4.33)	2.46*** (1.98 3.07)
1.FH#1.HTM	24.73*** (17.55 34.84)	11.11*** (8.72 14.17)
**Measures of interaction on the additive scale (P-value, 95% CI)**		
**RERI**	**14.50**; 0.001 (6.18 22.81)	**4.91**; 0.000 (2.33 7.49)
**AP** ^$^	**0.59**; 0.000 (0.44 0.73)	**0.44**; 0.000 (0.30 0.58)
**S**	**2.57**; 0.000 (1.79 3.68)	**1.94**; 0.000 (1.47 2.56)

*p < 0.05, **p < 0.01; ***p < 0.001; Ref, reference category; FH, family medical history of diabetes; HT, hypertension; HTM, hypertensive individuals who were on medication

Interaction exists if RERI!= 0 or AP!= 0 or S!= 1

Model 1: Unadjusted model; Model 2: Adjusted for age, sex, education, working, marital status, residence, MPCE, religion, caste, region, physical inactivity, smoking, chewing tobacco, alcohol consumption, and body mass index (BMI), ADL, IADL, high cholesterol

RERI, Relative excess risk due to interaction; AP, Attributable proportion; AP^$^, the attributable proportion, has been presented in the result as a percentage after multiplied by 100; S, Synergy index

In the additive model, the interaction effects between family history of diabetes and hypertension were found to be significantly positive, which demonstrates that the combined effect of two exposures (family history of diabetes and hypertension) is larger than the sum of the individual effects on the diagnosis of diabetes.

### Interaction effect between family medical history and hypertension on diabetes

Our study findings show that the adjusted odds ratio (AOR) of the joint effect between family medical history of diabetes and hypertension on the diagnosis of diabetes was 9.28 (95% CI: 7.51–11.46). In the adjusted model, the relative excess risk due to interaction (RERI) was 3.5 (95% CI: 1.52–5.47), which indicates that there is a significant positive interaction between family history and hypertension on the diagnosis of diabetes. The attributable proportion due to interaction (AP) value was 37% (0.37; 95% CI: 0.22–0.51), which suggests that a significant proportion of individuals with diabetes in the population can be attributed to the interaction between family medical history of diabetes and hypertension. The synergistic effect index (S) was 1.69 (95% CI: 1.31–2.18), further supporting a significant synergistic effect.

Furthermore, our findings further support the interaction effect on all the three measures based on hypertensive individuals who were taking medication. In the adjusted model, the RERI, AP and S values for diabetes were 3.79 (95% CI: 1.58-6.01), 40% (0.40; 95% CI: 0.25–0.55), and 1.81 (95% CI: 1.37–2.4) respectively, which indicates that there is a significant positive interaction between family history of diabetes and hypertension (taking antihypertensive medication) on the diagnosis of diabetes.

All the three measures of interaction, the RERI, AP and S, show significant positive interaction on the additive scale demonstrating consistent results in all the models (self-reported hypertension, and hypertensive individuals receiving medication) even after adjustment with potential confounding factors.

### Interaction effect between family medical history and hypertension on the diagnosis of diabetes (taking medication for diabetes)

The interaction effect of family medical history of diabetes and hypertension is further supported by selecting the individuals with diabetes who were taking medications.

#### Interaction effect between family medical history and hypertension on the diagnosis of diabetes

The results show that the adjusted odds ratio (AOR) of the joint effect between family medical history of diabetes and hypertension on the diagnosis of diabetes was 8.5 (95% CI: 6.82–10.61). In the adjusted model, the RERI, AP, and S for diabetes were 3.24 (95% CI: 1.46–5.03), 38% (0.38; 95% CI: 0.24–0.52), and 1.76 (95% CI: 1.34–2.3) respectively, which indicates that there is a significant positive interaction between family history of diabetes and hypertension on the diagnosis of diabetes.

#### Interaction effect between family medical history and hypertensive individuals taking antihypertensive medication on the diagnosis of diabetes

Furthermore, our findings further support the interaction effect on all the three measures based on hypertensive individuals who were taking medication. In the adjusted model, the RERI, AP and S values for diabetes (with medication) were 4.91 (95% CI: 2.33–7.49), 44% (0.44; 95% CI: 0.30–0.58), and 1.94 (95% CI: 1.47–2.56) respectively, which indicates that there is a significant positive interaction between family history of diabetes and hypertension on the diagnosis of diabetes. The findings present that the combined effect of family medical history of diabetes and hypertension on the risk of developing diabetes is greater than the sum of their individual effects.

All the three measures of interaction, the RERI, AP and S, show significant positive interaction on the additive scale demonstrating consistent results in all the models (self-reported hypertension, hypertensive individuals receiving medication) even after adjustment with potential confounding factors. Consequently, indicates that the combined effect of family history of diabetes and hypertension is more than the sum of the individual effects on the risk of developing diabetes among older adults aged 45 years and above in India.

## Discussion

The current study shows the interaction effect of family history of diabetes with hypertension on the diagnosis of diabetes. The study demonstrated a significant positive interaction on an additive scale between family history of diabetes and hypertension on diabetes as observed through all three measures (RERI, AP and S), even after adjustment with potential confounding factors, supported by different models of sample selection. The findings present that the combined effect of family medical history of diabetes and hypertension on the risk of developing diabetes is greater than the sum of their individual effects.

Our finding shows that the prevalence of diabetes was more than three times higher among individuals with family medical history of diabetes compared with those without family medical history. A prior study showed that the prevalence of diabetes was 30% for individuals with a high familial risk of diabetes, 14.8% for those with a moderate risk, and 5.9% for those with an average risk [6]. The more parents a person has with diabetes, the more likely they are to develop diabetes themselves. According to a previous study, the prevalence of diabetes increased significantly with the number of affected parents [4]. Our finding further shows that individuals with family history of diabetes had nearly 2.5 times higher odds of having diabetes than individuals without family history. A previous study revealed that a family medical history was a significant predictor for diabetes [4]. A previous study further showed that individuals with a moderate familial risk of diabetes had 2.3 times higher odd of having diabetes than those without a family history of diabetes. Individuals with a high familial risk had 5.5 times higher odds of having diabetes [6].

Additionally, our results demonstrate that individuals with parental and sibling medical history of diabetes had nearly 2.8 and 2.2 times higher odds of having diabetes, respectively, than those without parental and sibling history. In the Framingham Offspring Study, it was observed that the presence of a parental or sibling history with diabetes was associated with a 3.4-fold increased risk of diabetes, and this risk further escalated to 6.1-fold when both parents were affected [38]. Consistently, a prior study revealed that the presence of diabetes in one’s spouse was associated with an odds ratio (OR) of 2.32 for the occurrence of either diabetes or prediabetes, even after adjusting for BMI [39]. Moreover, our findings present that individuals with father, mother, brother, sister medical history of diabetes had significantly 3.29, 2.16, 2.37, and 2.40 times higher odds of having diabetes, respectively compared with those without family history.

Our results show that the prevalence of diabetes was more than four times higher among individuals with hypertension compared with those without hypertension. In addition, individuals with hypertension had 3.95 times higher odds of having diabetes than those without hypertension. In a prospective cohort study, it was observed that the incidence of type 2 diabetes was nearly 2.5 times higher among individuals with hypertension than those without hypertension [16].

Interaction refers to a condition in which the effect of one exposure on an outcome is modified by the level of another exposure [34, 35, 40]. Our findings present that the prevalence of diabetes was significantly higher among individuals with hypertension with family history of diabetes (46.6%) than those without the coexistence of family history of diabetes and hypertension (9.9%) individuals with hypertension and without a family history of diabetes (22.7%), and individuals with family history of diabetes and without hypertension (16.5%).

Furthermore, our findings show that, in the additive model, the interaction effects between family history of diabetes and hypertension were found to be significantly positive, which demonstrates that the combined effect of two exposures is larger than the sum of the individual effects of family history of diabetes and hypertension on the diagnosis of diabetes. Interestingly, it was observed that all the three measures of interaction, the RERI, AP and S, show significant positive interaction effect on the additive scale demonstrating consistent results for all the models (self-reported hypertension, and hypertensive individuals with medication) in two different samples (self-reported diabetes and individuals with diabetes receiving medication), even after adjustment with potential confounding factors. Moreover, the results suggest that a significant proportion of individuals with diabetes in the population can be attributed to the interaction between family medical history of diabetes and hypertension. Existing literatures demonstrated that the development of hypertension in people with diabetes not only adds complexity to treatment approach and escalates healthcare expenditure but also substantially elevates the risk of macrovascular and microvascular complications [41, 42]. Diabetic nephropathy represents a prevalent complication among individuals with diabetes and its risk is significantly higher in people with hypertension [43].

### Implications for policy, practice and future research

Beyond its role as a risk factor for type 2 diabetes, a familial history of diabetes was found to be associated with increased risk awareness and the adoption of lifestyle behaviors that reduces the risk of developing diabetes [8]. Similarly, in a previous study, it was observed that family history exhibited a stronger association with the perception of changing their behaviour to reduce their risk of type 2 diabetes compared to genetic risk testing [44]. Additionally, a prior study revealed that people with a genetic predisposition to diabetes can reduce their risk of developing the disease by making healthy lifestyle choices [45]. The researchers found that people with a high genetic risk of diabetes who adopted the healthy lifestyle behaviors had a risk of developing diabetes that was similar to people with a low genetic risk of diabetes [45]. Moreover, a recent study demonstrated that blood pressure lowering is an effective strategy for preventing the onset of new-onset type 2 diabetes. Across all trials, a 5 mmHg reduction in systolic blood pressure reduced the risk of type 2 diabetes by 11% [46].

The findings of the study also have potential implications for the routine clinical management of individuals with diabetes mellitus. Therefore, it is advisable to closely monitor individuals with blood pressure values approaching the upper limit of the normal range, particularly if they possess a positive family history of diabetes. It is crucial to note that the onset of hypertension in individuals with diabetes is accompanied by a noteworthy escalation in both macrovascular and microvascular risk [42, 47]. Consequently, concerted efforts should be directed toward the prevention of blood pressure elevation in these patients. A family history of diabetes can be a valuable tool to identify and target people at high risk of undiagnosed diabetes among hypertensive individuals, which could help to develop more targeted and effective approaches to earlier diagnosis and treatment and improved health outcomes. Additionally, a positive family history of diabetes could be used to improve risk counselling, especially when encouraging people to make lifestyle changes. Healthcare providers can also use family health history to identify people who may benefit from genetic risk testing.

Implementing a tailored intervention strategy through community health workers can improve adherence to evidence-based medication and promote healthy lifestyle practices in diabetes patients, leading to improved clinical risk markers. Incorporating trained community health workers in reaching high risk population may strengthen secondary prevention particularly in specific subgroups or community settings. Research on the effectiveness of lifestyle interventions for individuals with diverse health backgrounds, including family medical history, high blood pressure, and prediabetes, is essential. Investigating the long-term consequences of lifestyle interventions in these individuals, tracking changes in the health system and policy to support and sustain lifestyle interventions is equally imperative. There is critical need for further exploration of biomarkers that can enhance risk prediction and drug response in diabetes management, facilitating tailored primary and secondary prevention strategies more effectively. Further development of clinical decision support systems for team-based diabetes care and emphasizing shared decision-making and patient-centered care are crucial for advancing diabetes prevention efforts, with a focus on population-specific research and interventions to enhance overall effectiveness.Limitations and strengths.

The present study is a cross-sectional survey design and based on the first wave of the LASI data, thus cannot establish a causal relationship. Additionally, the study relies on self-reported information, which may subject to reporting bias. The current study revealed that more than 80% of the respondents were receiving medication for diabetes, which minimizes the recall bias. It is important to recognize these limitations while interpreting the findings of this study. Despite these limitations, the current study has potential strengths. In LASI, respondents were asked additional question concerning diagnosing physician, the date of diagnosis and if currently taking medication to those who reported being diagnosed with a disease by a medical professional. This study provided the estimates of interaction effect on additive scale for the two models of hypertension (self-reported hypertension, and hypertensive individuals receiving treatments) in two different samples (self-reported diabetes and individuals with diabetes receiving medication). Moreover, this is the first population-based study with a large sample size that explored the interaction effect of family medical history of diabetes with hypertension on diabetes in LMIC setting, especially in India. Further exploration and validation of the observed additive interactions require additional research employing rigorous study designs, such as prospective cohort studies or randomized controlled trials.

## Conclusions

The study findings strongly suggest that the interaction between family history of diabetes and hypertension has a significant and positive effect on the risk of diabetes and demonstrates the synergistic effect where the combined effect of these two exposures is greater than the sum of their individual effects. The results underscore the importance of considering both family medical history of diabetes and hypertension when assessing diabetes risk and designing preventive strategies or interventions.

## Data Availability

The datasets used and/or analysed during the current study are available in the repository of the Gateway to Global Aging Data (https://g2aging.org/ ).

## Abbreviations

RERI: Relative Excess Risk due to Interaction
AP: Attribution Proportion
S: Synergy index
HT: Hypertension
CAD: Coronary Artery Disease
CHD: Coronary Heart Disease
AOR: Adjusted Odds Ratio
CI: Confidence Interval
